# Relationship between body weight and the increment in serum brain-derived neurotrophic factor after oral glucose challenge in men with obesity and metabolic syndrome: A prospective study

**DOI:** 10.1097/MD.0000000000005260

**Published:** 2016-10-28

**Authors:** I-Te Lee, Jun-Sing Wang, Chia-Po Fu, Shih-Yi Lin, Wayne Huey-Herng Sheu

**Affiliations:** a Division of Endocrinology and Metabolism, Department of Internal Medicine, Taichung Veterans General Hospital, Taichung; b School of Medicine, National Yang-Ming University, Taipei; c School of Medicine, Chung Shan Medical University; d Center for Geriatrics and Gerontology, Taichung Veterans General Hospital; e Institute of Medical Technology, National Chung-Hsing University, Taichung, Taiwan.

**Keywords:** brain-derived neurotrophic factor, exercise, metabolic syndrome, weight reduction

## Abstract

Brain-derived neurotrophic factor (BDNF) plays a role in energy homeostasis. However, the postprandial BDNF change has not been well investigated. We hypothesized that the BDNF increment after oral glucose challenge is associated with body weight.

To address this possibility, man adults with obesity in conjunction with metabolic syndrome were compared with normal weight controls at baseline in the initial cross-sectional protocol. The obese subjects then underwent a 12-week program for body-weight reduction in the prospective protocol. The area under the curve (AUC) of serum BDNF was recorded during a 75 g oral glucose tolerant test and the BDNF AUC index was defined as [(AUC of BDNF) − (fasting BDNF^∗^2 hours)]/(fasting BDNF^∗^2 hours).

A total of 25 controls and 36 obese subjects completed the study assessments. In the cross-sectional protocol, the BDNF AUC index was significantly higher in the obese subjects than in the controls (9.0 ± 16.5% vs. − 8.0 ± 22.5%, *P* = 0.001). After weight reduction (from 97.0 ± 12.5 kg to 88.6 ± 12.9 kg, *P* < 0.001), the percentage change of body weight was significantly associated with the BDNF AUC index after the study (95% CI between 0.21 and 1.82, *P* = 0.015). Using 6% weight reduction as a cut-off value, a larger weight reduction was able to reliably predict a negative BDNF AUC index.

In conclusion, a high BDNF AUC index was observed for obese men in this study, whereas the index value significantly decreased after body-weight reduction. These findings suggest that postprandial BDNF increment may be associated with obesity.

## 1 Introduction

Metabolic syndrome, a clustering of cardiovascular risks, is associated with cardiovascular events and increased mortality.^[^1 2^]^ Because of the high prevalence, metabolic syndrome has become a heavy burden on global health.^[^3 4^]^ Obesity in turn is considered to represent the core pathogenesis of metabolic syndrome.^[^5–7^]^ Several meta-analysis studies have reported that obesity was associated with a higher risk of cardiovascular death than normal weight in the metabolically healthy population. Furthermore, metabolic syndrome was shown to contribute an additional risk of cardiovascular events in subjects with obesity categorized based on body mass index (BMI).^[^8–10^]^ Therefore, both BMI and metabolic syndrome should be considered for the assessment of cardiovascular risks.^[^10 11^]^

Weight reduction was found to result in an improvement of cardiovascular risks among subjects with obesity following short-term intervention of less than 1 year.^[^12–14^]^ However, the effect of weight reduction on the central nervous system remains controversial. An average weight reduction of 8.4 kg was shown to result in a significant improvement of depressive symptoms after a 6-month program of caloric restriction.^[14]^ Additionally, Horie et al^[15]^ reported that an average BMI reduction of 1.7 kg/m^2^ led to a significant cognitive improvement in elderly subjects with obesity and mild cognitive impairment after a 12-week program of caloric restriction. However, Bryan et al^[16]^ reported that an average weight reduction of 7.9 kg resulted in little impact on cognitive performance in overweight women after a 12-week program of diet restriction. Therefore, the interaction between energy homeostasis change and central nervous system response should be further investigated.

Brain-derived neurotrophic factor (BDNF), a member of the neurotrophin family, plays an important role in neural protection and synaptic activity.^[^17 18^]^ For example, upon BDNF binding, tyrosine receptor kinase B can activate the mitogen-activated protein kinase signaling pathway, which is associated with central nervous functions.^[^19 20^]^ BDNF can also be detected in peripheral blood and the circulating BDNF level has been found to reflect the level in the central nervous system.^[^21–23^]^ Notably, low circulating BDNF levels are associated with several central nervous disorders, such as depression, psychosis, and cognitive impairment.^[^24–26^]^

BDNF is also associated with energy homeostasis.^[27]^ It has been reported that high fasting serum BDNF concentrations were observed in women with obesity.^[^28 29^]^ Furthermore, circulating BDNF also positively correlated with the risk factors of metabolic syndrome.^[^29 30^]^ However, a contrary finding has also been reported wherein fasting BDNF concentrations were decreased in subjects with obesity upon cross-sectional assessment.^[31]^ Thus, the relationship between obesity and fasting BDNF remains largely controversial.^[32]^

BDNF, with its anorexigenic characteristics, has been suggested to increase within 1 hour after food intake.^[33]^ However, the effects of body weight on the alterations of serum BDNF concentration after glucose intake have not been well studied. To better understand the alterations in serum BDNF after glucose loading in subjects with obesity, we assessed the serum BDNF concentrations during a 2-hour glucose tolerance test (OGTT) between subjects with obesity and controls. In addition, we also prospectively examined the serum BDNF concentrations during OGTT in subjects with obesity following a body-weight reduction program.

## 2 Methods

### 2.1 Subjects

This study was conducted in Taichung Veterans General Hospital. The inclusion criteria consisted of adult men with ages between 20 and 75 years, obesity with BMI greater than 27 kg/m^2^,^[34]^ and meeting the criteria of metabolic syndrome as defined by the International Diabetes Federation.^[35]^ The exclusion criteria were: taking anti-diabetic medications, taking medications for schizophrenia or bipolar disorder, taking medications that caused changes in body weight, such as systemic steroids, changes in medications for hypertension, hyperlipidemia, anti-platelet, or anti-inflammation in the past month, endocrine diseases such as thyroid or adrenal disorders, acute or chronic renal diseases with serum creatinine levels greater than 200 mmol/L, severe systemic diseases such as malignant or immune disorders, and addiction to alcohol or drugs. The purposes of these exclusion criteria were to limit the possibility that body weight and BDNF levels were affected by underlying disease or medication use.

### 2.2 Assessments

The study comprised both case-control and prospective body-weight reduction assessments in the subjects with obesity. At baseline, age-matched controls with a BMI less than 24 kg/m^2^ and without metabolic syndrome were enrolled from man volunteers. All study subjects underwent the 75 g OGTT and blood samples were collected at fasting and at 30, 60, 90, and 120 minutes after glucose loading. In follow-up assessment, a 12-week program for weight-reduction was applied to the subjects with obesity. In the weight reduction program, subjects maintained a 1200 kcal/day diet containing 55% carbohydrate, 15% protein, and 30% fat. Subjects also attended 8 classes of moderate-strength aerobic exercise under the supervision of trained instructors. No medications were allowed to be changed during the study. The 75 g OGTT was assessed again after the 12-week program.

Serum concentrations of glucose and BDNF were assessed at 0, 30, 60, 90, and 120 min during OGTT. Fasting blood samples were also analyzed for insulin, lipids, liver enzymes, creatinine, and C-reactive protein (CRP). This study was approved by the Institutional Review Board of Taichung Veterans General Hospital and written informed consent was provided by all participants (NCT number: 01065753).

**Figure 1:**
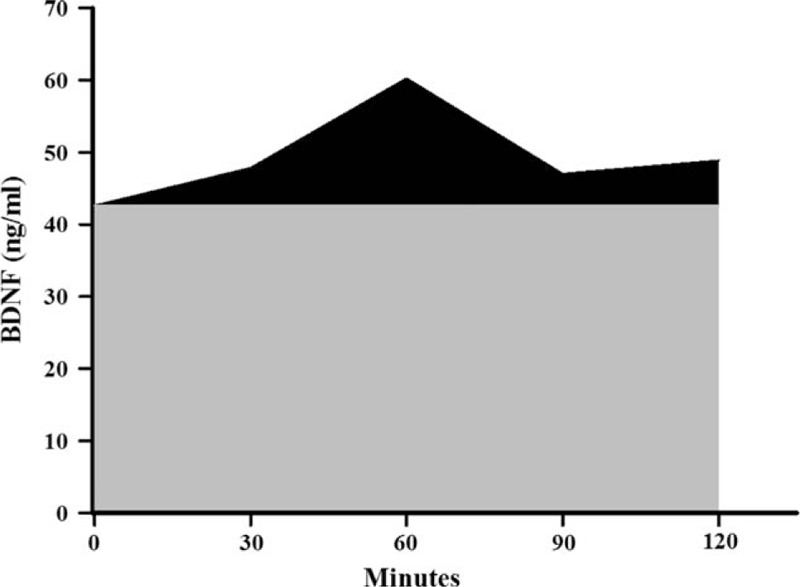
Description of the calculation used for determining the BDNF AUC index during 2-hour oral glucose tolerance test. Black area: the increase area under the BDNF curve during OGTT, Gray area: the area under the fasting BDNF value, BDNF AUC index: the ratio of black area to gray area. AUC = area under the curve, BDNF = brain-derived neurotrophic factor.

The serum concentration of glucose, triglyceride, cholesterol, and creatinine were detected using commercial kits (Beckman Coulter, Fullerton, CA, USA). Insulin and high-density lipoprotein (HDL) cholesterol were also detected using commercial kits (Roche Diagnostics GmbH, Mannheim, Germany). The homeostasis model assessment of insulin resistance (HOMA-IR) index was calculated as fasting insulin (μIU/mL)^∗^fasting glucose (mmol/L)/22.5.^[36]^ CRP was detected using an immunochemical assay (Good Biotech Corp., Taichung, Taiwan). Serum BDNF was detected using a commercial immunoassay kit (R&D Systems, Minneapolis, MN), for which the mean intra-assay and inter-assay CVs were 4.1% and 9.0%, respectively. The area under the curve (AUC) of BDNF during OGTT was calculated. We defined the BDNF AUC index as [(AUC of BDNF) − (fasting BDNF^∗^2 hours)]/(fasting BDNF^∗^2 hours) (Fig. 1).

### 2.3 Statistical analyses

All data are presented as the means ± standard deviation (SD). Differences in continuous variables at baseline were analyzed using an independent *t* test. A chi-square test was used to assess the differences in categorical variables. A paired *t* test was used to compare the difference prior to and following body-weight reduction. Multivariate linear regression analysis was employed to analyze the effect of obesity or body-weight change on BDNF. Statistical analysis was performed using SPSS 19.0 (IBM, Armonk, NY).

## 3 Results

### 3.1 Case-control assessments at baseline

**Figure 2:**
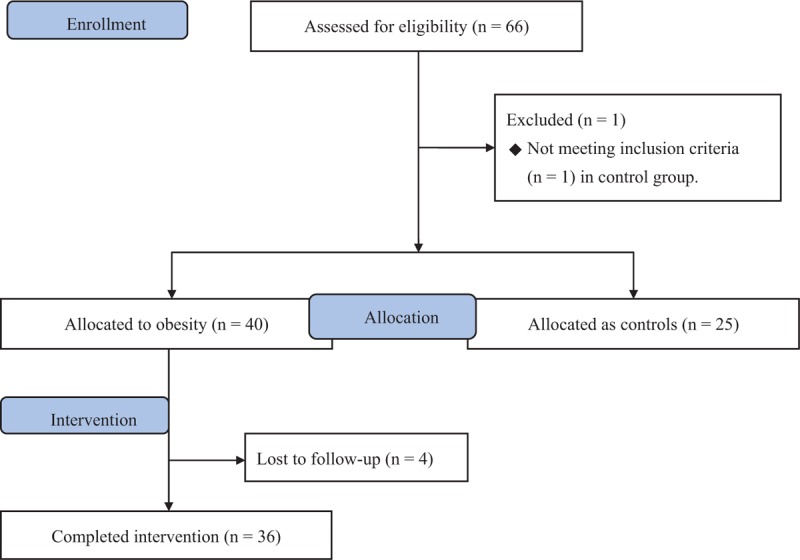
Flow diagram of enrollment of study subjects.

A total of 36 subjects with obesity and 25 controls were enrolled and completely assessed in this study (Fig. 2). There were 2 subjects with obesity and 1 control diagnosed with diabetes at baseline according to the post-challenge glucose level at 120 minutes (PC 2 hours glucose). The clinical characteristics of the obesity and control groups are shown in Table 1. BMI, blood pressure, fasting glucose, triglyceride, HOMA-IR, and CRP were significantly higher in the obesity group than in the control group (all *P* values lower than 0.001). HDL cholesterol was significantly lower in the obesity group than in the control group (*P* < 0.001).

**Table 1:**
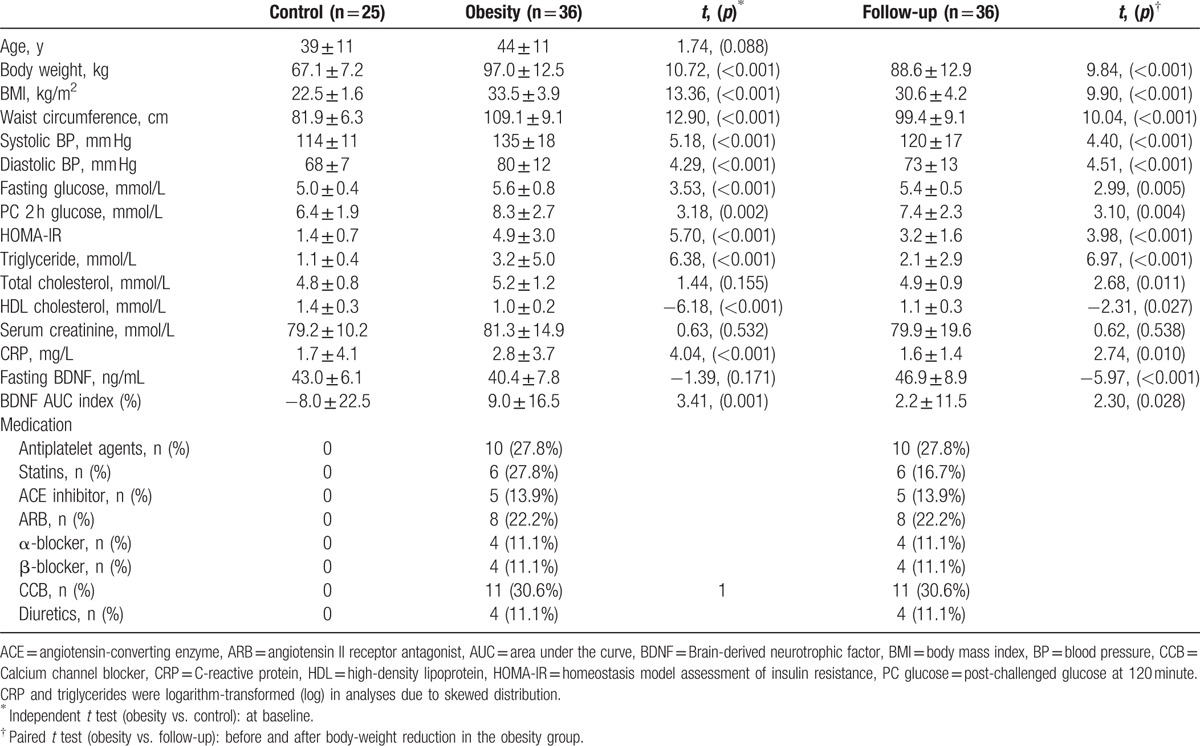
Clinical data of the subjects before and after the study.

**Figure 3:**
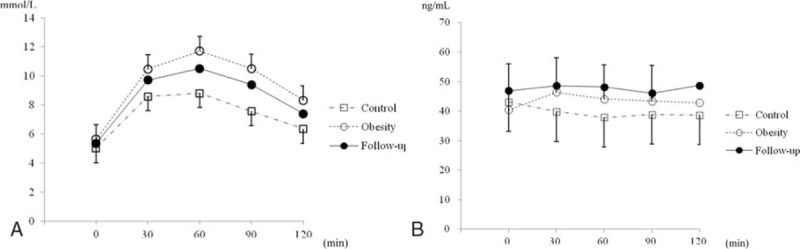
The glucose (A) and BDNF (B) concentrations at 0, 30, 60, 90, and 120 minutes during OGTT. BDNF = brain-derived neurotrophic factor, OGTT = oral glucose tolerance test.

The mean fasting BDNF concentration in the obesity group was not significantly different from that in the control group (40.4   7.8 vs. 43.0 ± 6.1 ng/mL, *P* = 0.171). The glucose and BDNF concentrations during OGTT are shown in Fig. 3. The BDNF AUC index was significantly greater in the obesity group than in the control group (9.0 ± 16.5 vs. −8.0 ± 22.5, *P* = 0.001). Using multivariate regression analysis, obesity was identified as an independent factor for a high BDNF AUC index after adjustment for age, CRP, and PC 2 hours glucose (Table 2).

**Table 2:**
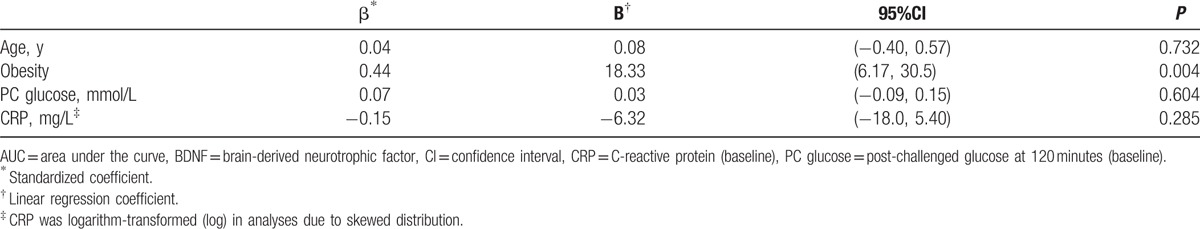
Multivariate association between obesity on the BDNF AUC index at baseline cross-sectional assessment.

### 3.2 Longitudinal effect of body-weight reduction

After the 12-week program for weight reduction, body weight was significantly decreased from 97.0 ± 12.5 kg to 88.6 ± 12.9 kg among the 36 subjects with obesity (*P* < 0.001). In addition, the components of metabolic syndrome, total cholesterol, and CRP were significantly improved after the study. Furthermore, the BDNF AUC index was significantly lower after body-weight reduction than that prior to the study (from 9.0 ± 16.5 to 2.2 ± 11.5, *P* = 0.028) (Table 1). Using multivariate regression analysis, the percentage change of body weight was significantly associated with the BDNF AUC index at the end of the study (Table 3).

**Table 3:**
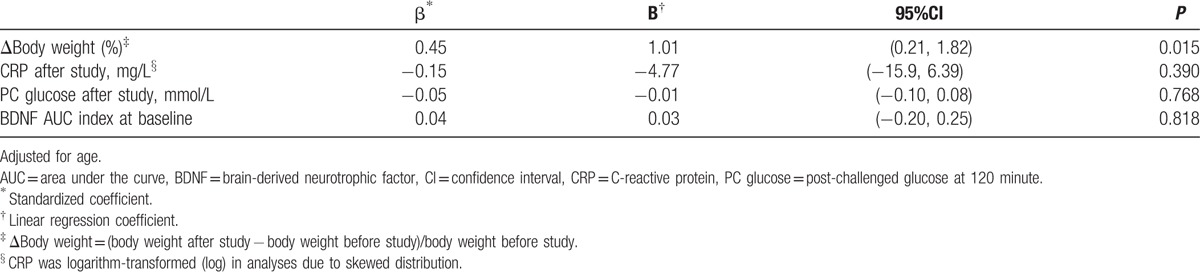
Multivariate association between change of body weight and BDNF AUC index at the end of study.

**Figure 4:**
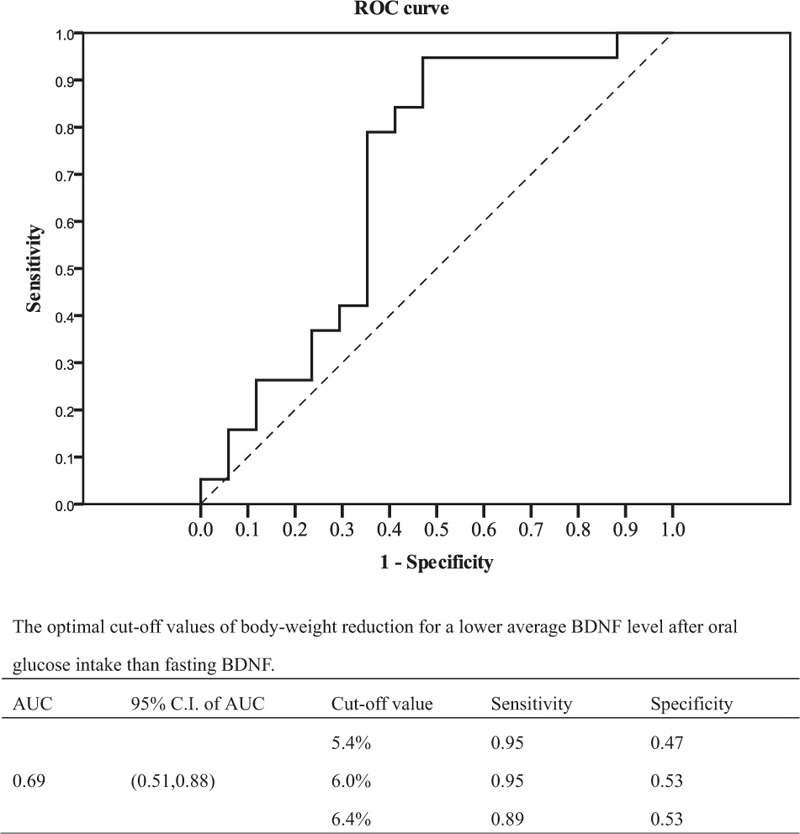
Receiver operating characteristic (ROC) analysis curve and cut off level of body-weight reduction for negative BDNF AUC index after glucose intake. AUC = area under the curve, BDNF = brain-derived neurotrophic factor.

As a greater reduction in body weight was associated with a lower BDNF AUC index following the study, the percentage of body-weight change was assessed by receiver operating characteristic (ROC) analysis to differentiate a negative BDNF AUC index (i.e., BDNF AUC less than fasting BDNF^∗^2 hours) at the end of the study. The AUC in the ROC analysis was 0.69 (95% confidence interval, 0.51–0.88). The cut-off value of 6% weight reduction yielded a sensitivity of 95% and specificity of 53% for a negative BDNF AUC index in the subjects with obesity after the study (Fig. 4).

## 4 Discussion

In the present study, we reported a greater increment of serum BDNF concentration as demonstrated by the BDNF AUC index after glucose intake in men with obesity and metabolic syndrome than in controls. Previously, serum BDNF had been associated with an inhibitory effect on food intake and an enhancement of energy expenditure.^[27]^ Accordingly, an increase in BDNF after glucose intake was, therefore, assumed to play a certain role in body-weight balance.^[21]^ However, fasting BDNF concentration was not significantly associated with body-fat mass, and did not significantly correlate with body-weight change in our previous studies.^[^32 37^]^ As BDNF concentration responded to the stimulation of food intake, our findings together suggested that the postprandial elevation of circulating BDNF, but not the fasting level, might be responsible for obesity in men.

Furthermore, a longitudinal reduction in body weight would be expected to reduce the circulating anorexic peptides and this alteration might act as a barrier against long-term diet restriction.^[38]^ Comparable to the results obtained from other anorexic peptides in a previous report from Sumithran et al,^[38]^ our findings also showed that the BDNF AUC index decreased following body-weight reduction in subjects with obesity and metabolic syndrome. The BDNF AUC index was associated with the reduced percentage of body weight, independent from the baseline BDNF AUC index. Notably, the mean serum BDNF concentration within 2 hours after 75 g glucose intake was lower than that in the fasting status once weight reduction reached ≥6%.

In addition, it has been reported that the fasting serum BDNF level was inversely correlated with the risks of cardiovascular diseases.^[^32 39^]^ Alternatively, BDNF is predicted to enhance the synaptic innervation to sympathetic neurons and induce sympathetic activation.^[40]^ A high BDNF concentration has also been associated with coronary oxidative stress^[41]^ and BDNF could be detected in coronary lesions within several hours after ischemia.^[42]^ Thus, the postprandial increment of BDNF might be associated with cardiovascular diseases and postprandial angina.^[^43–45^]^ In the present study, a high serum BDNF level was observed within 2 hours of OGTT in subjects with obesity and metabolic syndrome. The abated postprandial BDNF observed after short-term body-weight reduction might, therefore, reflect cardiovascular improvement. However, neither cardiovascular events nor cognitive function were shown to significantly improve in patients with type 2 diabetes after a long-term lifestyle intervention in the Look AHEAD study.^[^46 47^]^ Similarly, cardiovascular death was not significantly reduced by a 4-year lifestyle intervention in overweight subjects in the Finnish Diabetes Prevention Study after a 10-year follow up.^[48]^ Nor, in the Da Qing Diabetes Prevention Study, was the cardiovascular death significantly reduced by a 6-year lifestyle intervention in first 20 years,^[49]^ although the reduction in cardiovascular death become significant after 23 years of follow up.^[50]^

In the present study, the BDNF AUC index was not associated with either fasting or PC 2 hours glucose levels. Suwa et al^[28]^ also reported that serum BDNF was not significantly associated with glucose levels during OGTT. Therefore, the alteration in circulating BDNF levels after oral glucose intake might not be directly responsive to hyperglycemia, suggesting that the source of cerebral BDNF output might also play an important role.^[^31 51^]^ For example, in animal studies, the expression of BDNF in the brain has been shown to be induced by vagal afferents or humoral interactions.^[^33 52^]^

There were some limitations in the present study. First, we did not directly assess the mechanism underlying the high BDNF AUC index in cardiovascular disease. Many factors in addition to oxidative stress are considered to be involved in the association between BDNF and cardiovascular risks.^[41]^ It has also been reported that the anorexic effect of BDNF might correlate with the levels of several cerebral peptides such as leptin or cholecystokinin, which also have peripheral effects on cardiovascular diseases.^[^33 53 54^]^ Second, all subjects with obesity underwent both diet restriction and exercise promotion. We previously reported that an increase in muscle power after aerobic exercise promotion, independent of body-weight change, could increase fasting serum BDNF.^[37]^ However, in the current study we could not determine which type of life-style change provided the greater effect in reducing the BDNF AUC index. Third, the long-term effect of weight reduction was not analyzed in the present study. Sumithran et al^[38]^ has reported that a reduction in anorexic signals might not revert during one year of follow up. Therefore, examination of the BDNF AUC index should be pursued in future long-term studies.

In conclusion, an elevated BDNF AUC index but not fasting serum BDNF concentration was associated with obesity in our study. Weight reduction could result in a reduction in the BDNF AUC index within 12 weeks. The cut-off value of 6% reduction in weight was demonstrated to predict a negative BDNF AUC index in men with obesity. Therefore, the BDNF AUC index showed a good association with body weight. A more comprehensive understanding of the distinct roles and mechanisms of serum BDNF levels in various physiologic states will likely aid in the assessment and management associated cardiovascular risks and may lead to the identification of therapeutic targets.

## Acknowledgments

The statistical analysis was performed by the Biostatistics Task Force of Taichung Veterans General Hospital, Taichung, Taiwan.
